# Intensified training in adolescent female athletes: a crossover study of Greek yogurt effects on indices of recovery

**DOI:** 10.1080/15502783.2022.2044732

**Published:** 2022-03-22

**Authors:** Brandon J. McKinlay, Phillip J. Wallace, Shai Olansky, Stacey Woods, Nigel Kurgan, Brian D. Roy, Andrea R. Josse, Bareket Falk, Panagiota Klentrou

**Affiliations:** a Department of Kinesiology, Brock University, Ontario, St. Catharines, Canada; b Faculty of Applied Health & Community Studies, Sheridan College, Brampton, Ontario, Canada; c Centre for Bone & Muscle Health, Faculty of Applied Health Sciences, Brock University, Ontario, Canada; d School of Kinesiology & Health Science, York University, Toronto, Ontario, Canada

**Keywords:** Protein, carbohydrates, cytokines, creatine kinase, C-reactive protein

## Abstract

**Background:**

During a period of intensified exercise (e.g. training/identification camps), often undertaken by competitive youth athletes, the maintenance of muscle function and peak performance can become challenging due to an accumulation of fatigue. The provision of post-exercise dairy protein in adults has been previously shown to accelerate recovery; however, its efficacy in youth athletes is currently unknown. Therefore, the purpose of this study was to examine the effects of increased dairy protein consumption with plain Greek yogurt (GY) on performance and recovery indices during an intensified soccer training camp in adolescent female soccer players.

**Methods:**

Thirteen players (14.3 ± 1.3 years) participated in a randomized, double blinded, crossover design study where they received 3 servings/day of either GY (~115 kcal, 17 g protein, ~11.5 g carbohydrates) or an isoenergetic carbohydrate control (CHO, ~115 kcal, 0.04 g protein, ~28.6 g carbohydrates) during two 5-day soccer-specific training camps. Performance was assessed before and after each training camp. Fasted, morning, creatine kinase (CK), insulin-like growth factor-1 (IGF-1), C-reactive protein (CRP), interleukin 6 (IL6), interleukin 10 (IL10) and tumor necrosis factor-α (TNFα) were measured in plasma pre- and post-training.

**Results:**

Training led to decrements in counter-movement jump (*p* = 0.01), broad jump (*p* = 0.04) and aerobic capacity (*p = *0.006), with no effect of GY. A significant increase in anti-inflammatory cytokine IL10 was observed from pre- to post-training in GY (+26% [*p* = 0.008]) but not in CHO (*p* = 0.89). CRP and CK increased (+65% [*p* = 0.005] and +119% [*p ≤ *0.001], respectively), while IGF-1 decreased (−34% [*p ≤ *0.001]) from pre- to post-training with no difference between conditions.

**Conclusions:**

These results demonstrate that consumption of GY did not offer any added recovery benefit with respect to measures of performance and in the attenuation of exercise-induced muscle damage above that achieved with energy-matched carbohydrate in this group of young female soccer players. However, regular consumption of GY may assist with the acute anti-inflammatory response during periods of intensified training in adolescent athletes.

## 1. Background

Soccer is a team-sport characterized by aerobic and anaerobic components, encompassing both intermittent eccentric high-intensity (e.g. repeated sprints, acceleration/deceleration, jumping) and continuous aerobic activity. It has been estimated that during a match, a player can cover ~9–12 km of which ~2–3 km is at high-intensity [^1–3^]. Consequently, training programs for soccer players focus on developing aerobic and anaerobic components in an effort to improve players’ ability to perform and rapidly recover from high-intensity exercise [1]. These training programs are periodized throughout the competitive on-and-off season and are marked by short-term periods of intensified training (i.e. increased volume and intensity) in the form of training or talent identification camps [4]. The combination of intensified training and competition can lead to suboptimal-recovery and decrements in performance over time in adult [5], as well as in youth athletes [6]. In addition, periods of intensified exercise combined with eccentric movements inherent to soccer training and match play can lead to cumulative fatigue, which can delay recovery and the return of physical capacity [7]. This observation has been characterized by increased and sustained plasma concentrations of creatine kinase (CK), worsening muscle soreness, as well as chronic elevation of various markers of inflammation (i.e. cytokines and acute-phase proteins) and immune cells [5^,8–10^]. This prolonged inflammatory state is problematic for athletes during periods of increased daily energy expenditure [11], particularly if energy consumption does not match the energy demands (consumption < expenditure). Such a negative energy balance places additional strain on endogenous fuel stores potentiating fatigue further and delaying the return of physical capacity [11]. In addition to restoring physical capacity and maintaining homeostasis, energy and macronutrients must support healthy growth and development in youth athletes [12]. Therefore, nutritional interventions providing whole foods may provide substrates to attenuate the negative effects of intensified exercise in adolescent athletes.

The use of whole dairy foods (i.e. milk, yogurt and cheese) and/or isolated dairy proteins (i.e. whey and casein) may optimize recovery during a period of intensified training [13]. Specifically, milk contains essential amino acids, carbohydrates, fats and other nutrients that may expedite recovery through facilitating the repair, regeneration and replenishment processes [13]. Milk proteins have previously been observed to both stimulate muscle protein synthesis [14,15] and reduce the magnitude of protein breakdown [16] following acute resistance exercise. Additionally, consumption of whey protein following intense endurance exercise appears to attenuate the pro-inflammatory response (e.g. interleukin 6 [IL6] and tumor necrosis factor alpha [TNFα]) [17]. Likewise, consumption of whey protein following intense exhaustive exercise has been observed to increase anti-inflammatory cytokine concentrations (e.g. interleukin 10 [IL10]), possibly facilitating an earlier recovery response in both youth and adult athletes [18,19]. However, whether these beneficial physiological effects extend to prevent subsequent performance decrements during periods of intensified training is not clear.

Milk contains both whey and casein protein. The former is rapidly digested and absorbed, resulting in a large, but acute rise in amino acids. Casein protein, is slowly digested and absorbed, resulting in a more moderate, but sustained rise in amino acids [14,16]. This attribute of casein protein may make it the ideal supplement during periods of intensified exercise, as a prolonged positive protein balance could potentially attenuate muscle protein breakdown, thereby better preserving functional capacity. Greek yogurt (GY), a readily available casein-based, whole-food dairy-protein source, may be a viable recovery supplement during intensified training. Compared to milk, one serving of GY contains approximately double the protein content [20] and its solid form results in slower digestion compared with liquid recovery options [21]. Furthermore, while GY contains nutrients that are considered more beneficial for healthy bone development (e.g. calcium and phosphorus) [22], it also contains active cultured bacteria thought to enhance immunity [23]. While previous studies have investigated the effectiveness of GY to enhance resistance training-related adaptations (e.g. strength, muscle mass) in young men [24], to our knowledge, its effectiveness as a recovery aid during a short-term period of intensified exercise has not been examined.

The purpose of this study was to examine the effects of increased protein consumption by plain GY compared with an isoenergetic carbohydrate control on performance recovery, inflammation, and muscle damage, during a simulated soccer training camp in competitive adolescent female soccer players. It was hypothesized that regular provision of GY would result in better retainment of performance measures, an attenuated inflammatory response (i.e. lower concentration of pro-inflammatory or higher concentration of anti-inflammatory markers) and lower plasma CK levels compared with an isoenergetic carbohydrate control.

## 2. Materials and Methods

### Participants

**Figure 1.**
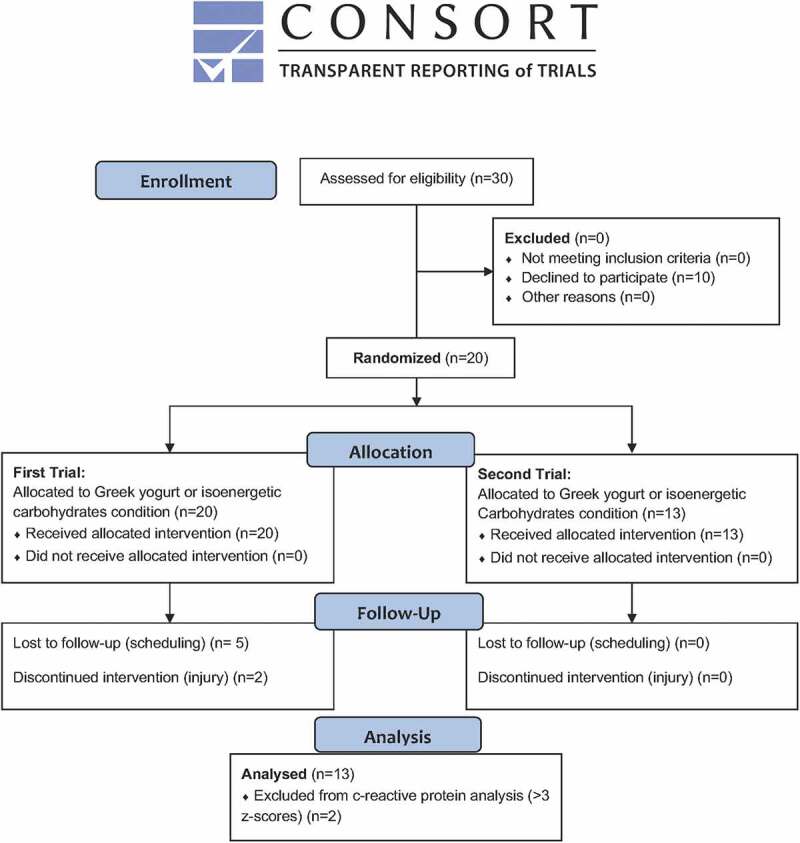
CONSORT flow Diagram.

The study was cleared by the Research Ethics Board of Brock University (REB# 18-289) and was registered at Clinicaltrials.gov (NCT03947801). Thirty competitive female soccer players were recruited to participate in this study. Of this original cohort, 10 players declined to participate and seven did not complete the crossover due to scheduling conflicts (*n* = 5) or sustained an injury (*n* = 2) unrelated to the study. In the end, 13 participants (14.3 ± 1.3 years of chronological age; 2.4 ± 0.7 years from age of peak height velocity [indicator of maturational status]) successfully completed the study protocol. Figure 1 shows the CONSORT diagram for the present study.

All participants and their parents/guardians received a thorough explanation of the study’s purpose, procedures, benefits, and potential risks, and written consent/assent was obtained from the participants and their respective parents/guardians prior to study commencement. To be eligible to participate in the study, all participants needed to be training at least 3 sessions per week and to have competed in travel soccer for a minimum of 5 years. Specifically, the included participants played competitively for 6.5 ± 2.2 years, trained on average 6.4 ± 0.8 h·week^−1^, competed in one match per week (1.5 h) and were outfield players. Additionally, participants were required to be free of any musculoskeletal injuries and medical conditions that would have prevented them from undergoing maximal exercise and were not currently taking any medications or nutritional supplements.

### Study Design

This study was a randomized, double-blinded, crossover trial. The protocol consisted of an information session, two identical training camps consisting of 5 consecutive days of intensified soccer training, differing only by nutritional intervention, and identical pre- and post-training session testing (see procedures and measurements below). Specifically, the intensified soccer training program increased the frequency (from 3 to 5 days), intensity (e.g. decreased rest duration, increased number of drills), and overall regular weekly training volume (from 6 to 10 h). All participants were post-menarcheal with regular menses but were not all tested in the same phase of their menstrual cycle. To control for potential menstrual effects, testing blocks were separated by 28 days, so that each participant was in the same phase of their menstrual cycle during the two training periods, which also allowed for an adequate wash-out and recovery period, during which participants resumed regular soccer activities.

### Experimental Procedures

#### Information session

The information session occurred ~3 weeks prior to the study’s commencement. During this session, participants and their parents/guardians signed the consent and assent forms and completed a medical screening questionnaire to report any injuries, allergies, and/or health-related conditions, along with information regarding menstruation (i.e. onset and regularity of cycle). Participants also completed a training and sport history questionnaire in which the level of competition, the number of years participating, training volume per week and perceived intensity of the given sport or training modality were described.

#### Pre- and Post-training testing

During the training camps, participants were tested twice: on the day before (pre) and day following (post) the 5-day soccer-specific training. Participants arrived at the University for pre- and post-training testing at 0800 h. Standing and seated height were measured using a portable stadiometer (SECA – 217, Canada), and recorded to the nearest 0.1 cm. Body-fat percentage, recorded to the nearest 0.1%, was estimated using bioelectrical impedance analysis (InBody520, Biospace Co. Ltd., Seoul, S. Korea), while simultaneously measuring body mass, recorded to the nearest 0.1 kg. A fasted venous blood sample was then taken from an antecubital vein. Participants then consumed a standardized breakfast, which included one granola bar, one muffin, one fruit (banana or apple) and a juice box or water (carbohydrates = 80 g or ~1.35 g·kg^−1^, protein = 5 g or ~0.08 g·kg^−1^ and fat = 20 g or ~0.34 g·kg^−1^, ~400–500 kcal). Participants were instructed to eat the same breakfast during the pre- and post-training testing sessions. At ~1045 h (~1.5 h postprandial), participants began a standardized dynamic warm-up (~15-20 min). Next, participants completed a battery of performance tests (see details below), following which they were provided a food frequency questionnaire (FFQ) to be completed with the assistance of a parent prior to the first training session, to determine habitual dietary patterns. During the period between the first pre-testing and the first training session, participants were randomly allocated to one of two experimental conditions, i.e. GY or the isoenergetic carbohydrate control (CHO), by an independent research assistant (described below), then crossed over to the alternative treatment following the washout period. Post-training testing was identical to pre-training testing sessions and occurred following the final day of the 5-day training protocol.

#### Simulated Soccer Training Camp

The simulated 5-day soccer-specific training was structured to mimic a heavy-volume, high-intensity training camp. Training sessions occurred at the same time each day (1800–2000 h), were matched for intensity, duration, and specific drills/exercises, and were administered by a certified technical soccer coach and knowledgeable training staff. Each session began with a 15 min dynamic warm-up followed by 90 min of soccer-specific training, ending with a 15 min cool-down. The 90 min of soccer-specific drills were performed at maximal effort and consisted of agility, sprinting and plyometric exercises as well as ball-handling, small-sided games (rondo) and shooting. Specifically, the work-to-rest ratios for drills for days 1, 3 and 5 were 1:1 or 1:2. On days 2 and 4, a longer rest interval was provided (1:3 and 1:4) for power-based drills (e.g. plyometrics). Following the completion of each training session, participants were asked to individually rate how hard the practice was using a standard rating of perceived exertion scale [^1–10^]. A mean rating was recorded for each 5-day period. The coach-to-participant ratio during all training sessions was 1:3.

### Nutrition Records

Participants were to self-report all food consumed in a 24h period for all 5-day of the training camp. In addition, participants were instructed to limit dairy consumption to 1 serving per day throughout the entire duration of the study. The food record was provided to each participant in a folder with a portion size sheet stapled to the inner flap. Each participant was individually educated with detailed instructions on how to complete the food records. Each day participants would complete and return the food record and be provided with a blank one for the subsequent 24-h period. To ensure similar diets between each training week, photocopies of the food records over the course of the first training week were provided to participants prior to the second week of training. All food records were analyzed using a diet analysis program (Food Processor, ESHA Inc., Salem, OR), by the same examiner for consistency. In addition, habitual dietary intake was assessed using a FFQ (NutritionQuest, Berkley, CA, USA), designed to assess dietary habits through a recall of foods eaten in the last 6 months.

### Nutritional Intervention

Participants consumed three servings of 160 g of plain GY (~115 kcals, 17 g protein, ~11.5 g carbs, 0 fat; active bacteria cultures: *S. thermophilus*, *L. bulgaricus*, *L. acidophilus*, *B. Llactis*) (Skotidakis Inc., St. Eugene, ON, Canada), or 30 g of isoenergetic CHO (~115 kcal, 0.04 g protein, ~28.6 g carbs, 0 fat) immediately following the training session, 1 h prior to bedtime, as well as one serving between breakfast and lunch on the subsequent day. The isoenergetic CHO serving was prepared daily, using a combination of 6 g fat-free vanilla Jell-O instant pudding (Kraft Heinz Canada, Toronto, ON, Canada), and 24 g maltodextrin (GLOBE Plus 15DE Maltodextrin 100,300, Ingredion, London, ON, Canada) mixed in water to match the color and consistency of GY. Both supplements were prepared by an independent research assistant to be served in clear containers and flavored with calorie-free vanilla syrup. The contents of the supplement were not divulged to the participants or the training staff. Participants were instructed to consume the supplements in addition to their habitual dietary intake. Participants were asked to report daily whether they consumed all intervention servings. Based on these reports, there was 100% compliance in supplement consumption over the duration of the study for both conditions. The true contents of both supplements were concealed until the completion of the study, where a post-experimental interview revealed 10 of the 13 participants could not differentiate between the GY and CHO supplements.

### Performance Test Battery

To measure progressive changes in physical performance following intensified training, participants performed a performance test battery before (pre-) and after (post-) the 5-day intensified training periods. Participants were instructed to refrain from exercise at least 48 h prior to the pre-training data collection for both visits. The performance tests included in the test battery were related to important skills involved in soccer such as speed, agility, change of direction, jumping, and power. Participants were familiarized with all performance testing protocols and procedures prior to assessment. Specifically, participants performed five maximal practice 20 m sprints, five maximal practice modified 5-0-5 (bi-directional), three maximal counter-movement jumps and three maximal broad jumps prior to assessment. However, participants were not familiarized with the beep-test as they regularly perform this assessment as part of their fitness testing with their respective clubs.

Speed was assessed using a 20-m sprint test using the Brower timing TCi-system (Brower Timing Systems, Draper, UT, USA). The TCi smart-start system block was placed at the starting line (0 m) and the Brower timing photocells were placed at 10 and 20 m. Participants were instructed to place their preferred foot on the starting line near the TCi smart-start block. Participants were instructed, ‘when ready, run as fast as you can through both gates, and do not slow down until you are past the last gate’, and were verbally encouraged throughout the duration of the sprint. Three maximal sprints, with ~3 min of rest between trials were performed, and the average of these sprints was taken for further analysis.

Agility was assessed using a modified 5-0-5 test with the Brower timing TCi system. The modified 5-0-5 agility test measured the ability of the participant to change direction. A starting line and a turnaround line were set up 5 m apart. Brower timing photocells were set up at the starting line. Participants were instructed to place their preferred toe on the starting line. When ready, participants were instructed to ‘run as fast as you can to the turn-around line, use one foot to break the plane of the turn-around line, plant, and sprint back through the starting line’. Two maximal trials were performed, one facing each direction, with ~3 min of rest between trials. The average of both trials was used for further analysis.

Vertical jump was assessed using the counter-movement jump (CMJ) measured by force plates (Hawkin Dynamics, Westbrook, ME, USA). Participants were instructed to step onto the force plates and to stand as still as possible (quiet phase). Upon a visual cue, with hands on hips the participant bent at the hip and knees into a squat position and immediately performed a vertical jump. The athlete was instructed not to bend their knees during flight time and to ‘stick’ the landing as best they could upon decent. Participants performed the CMJ three times, with approximately 3 min rest between trials. The average of all three trials was used for further analysis.

Horizontal lower body power was assessed using the standing broad jump (BJ), recorded as the farthest distance a participant could jump from a standing position. A starting line was taped along the floor, and a non-flexible open reel measuring tape was used to measure distance jumped. Participants were instructed to line the tips of their toes up with the beginning of the strip of tape. When instructed, participants propelled themselves forward as far as they could. Measurements were taken from the starting line to the heel of the foot that was closest to the starting line. Participants performed the BJ three times, with approximately 3 min rest between trials. The average of all three trials was used for further analysis.

Endurance was assessed using the 20 m shuttle run (beep-test), which required each participant to repeatedly run progressively faster 20 m intervals, until volitional fatigue. Each participant was required (on an auditory tone) to perform a 2 × 20 m run (down and back) in a predetermined time frame (second auditory tone). If the participant was unable to sustain the interval pace, i.e. if unsuccessful in reaching the starting line or turnaround line before the second auditory tone, they received a ‘warning’. Two consecutive ‘warnings’ resulted in test termination. The stage at which the test was terminated was then converted to distance in meters and used for further analysis.

### Blood Collection and Analysis

A total of 10 mL of blood was collected from an antecubital vein by a certified phlebotomist using a standard venipuncture technique during each pre- and post-testing session (total of 4 timepoints). Serum and plasma were collected using BD vacutainers. All the serum tubes were allowed to clot for 20 min at room temperature (23°C) before centrifugation at 4°C for 10 min at 1,405 *g*. All Ethylenediaminetetraacetic acid (EDTA) plasma tubes were centrifuged immediately at room temperature (23°C) for 10 min at 1,405 *g*. Following centrifugation, serum and plasma were immediately aliquoted into pre-labeled Eppendorf tubes, snap frozen in liquid nitrogen and subsequently stored at −80°C for future analysis.

Inflammatory cytokines were measured in plasma and analyzed using Multiplex magnetic bead kits (Milliplex EMD Millipore Corporation, US – Cat. #HSTCMAG-28SK). Creatine kinase was measured in plasma and analyzed using a commercially available reagent kit (Pointe Scientific INC, USA – Cat. #C7522) fitted onto a 96-well plate and normalized with purified creatine kinase (Sigma, CAN, Cat. 10127566001). Serum estradiol, plasma C-reactive protein (CRP), and plasma human insulin-like growth factor-1 (IGF-1) were analyzed using ELISA kits (Human Estradiol E2 kit, Abcam, CAN Cat. # ab108640; Human C-Reactive Protein/CRP Immunoassay, R&D systems, US, Cat. # DCRP00; Human IGF-1/IGF-1 Immunoassay, R&D systems, US, Cat. # DG100B). All analytes were measured in duplicates using multiple plates, with the average coefficients of variation (CV) estimated in-house. Specifically, the average inter-and intra-assay CVs for IL6, IL10 and TNFα were 5.3% and 2.2%, 6.6% and 3.9%, 7.6% and 3.0%, respectively. The average inter- and intra-assay CVs for CK were 5.0% and 8.2%, respectively. The average inter- and intra-assay CVs for estradiol were 5.2% and 8%, respectively. The average inter- and intra-assay CVs for CRP were 7.6% and 8.2%, respectively. Lastly, the average inter- and intra-assay CVs for IGF-1 were 7.5% and 6.4%, respectively.

### Statistical Analysis

Prior to study commencement, a priori sample size calculation was performed from a previous study in youth athletes [25], a minimum of 20 participants would be required for a power of 0.80 and a significance level set at 0.05. Hence, our original recruitment of 20 participants. However, only 13 participants successfully completed the crossover. All statistical analyses were performed using IBM SPSS version 25 for windows (SPSS Inc., USA). All continuous data are presented as mean ± 1 SE. Prior to analysis, all variables were tested for normality by visual inspection of histograms, *z*-scores (± 3) and by assessing the skewness and kurtosis (± 3). All variables, except CRP, met the assumptions of normality. For CRP, we identified two outliers with much higher levels (*z*-score >3) during the pre-training visit of week 1 compared to pre-training visit of week 2. These values were removed from the analysis. The remaining sample for CRP (*n* = 11), was then normally distributed.

A one-way ANOVA was used to examine differences in dietary intake between habitual and the two nutritional interventions at pre-training. Additionally, paired-sample *t*-tests were used to examine differences between the two nutritional interventions at pre-training in terms of physical characteristics, performance, and resting biochemical markers between training weeks. Differences in the ratings of perceived exertion between the two training periods were assessed using a Wilcoxon signed-ranked test and are presented as median and quartiles 1 and 3. A series of two-way repeated measures analysis of variances (RM-ANOVA) were then used to examine changes from baseline with main effects and interactions for the nutritional intervention (GY vs CHO) and time (pre- vs post-training) on performance and biochemical markers. In the event of a significant interaction, *post-hoc* comparisons were performed using a Bonferroni pairwise comparison. If the assumption of sphericity was violated, the Greenhouse Geisser correction factor was used. The results of the RM-ANOVA are reported as *F*, *p*, and partial *η^2^* (_p_*η^2^*). Cohen’s *d* effect sizes were calculated on absolute changes, where effects were considered small (0.2), medium (0.5), and large (>0.8) [26]. The significance level was set at *p* < 0.05.

## 3. Results

No differences were observed in height (*p* = 0.75), body mass (*p* = 0.47) and body fat % (*p* = 0.88) at pre-training between the two nutritional interventions (Table 1). Similarly, basal estradiol concentrations were not different (*p* = 0.91) between interventions (GY = 13.1 ± 3.1 pg·ml^−1^, CHO = 13.4 ± 3.4 pg·ml^−1^). Additionally, participants’ perceived level of exertion (*p* = 0.16) was similar between the two interventions (GY: 8 [8 – 9], CHO: 8 [8 – 9]).

**Table 1.** Participants’ physical characteristics, pre- and post-training during each nutritional intervention

	Greek Yogurt		Carbohydrate	
	Pre	Post	Pre	Post
Height (cm)	165.9 ± 1.4	165.9 ± 1.4	166.0 ± 1.5	166.0 ± 1.5
Body mass (kg)	59.1 ± 2.1	59.5 ± 2.1	59.3 ± 2.0	59.7 ± 2.1
Body fat (%)	22.2 ± 1.8	22.2 ± 1.5	22.2 ± 1.5	22.4 ± 1.5

Values are mean ± SE.

As presented in Table 2, our nutritional intervention was successful in creating two distinct conditions. Specifically, no differences in energy intake or fat were observed between GY and CHO, but protein intake was higher (*p* ≤ 0.001, *d* = 2.5) in the GY condition and carbohydrate intake was higher (*p* ≤ 0.001, *d* = 1.1) in the CHO condition (Table 2). When training diet (via food record) was compared to participants’ habitual diets (via FFQ), no differences were observed with respect to the energy intake and relative fat consumption. However, relative habitual protein and carbohydrate consumption were observed to be lower when compared to the training weeks, when GY (*p* ≤ 0.001, *d* = 2.2) and CHO (*p* = 0.003, *d* = 0.90) were consumed, respectively (Table 2).

**Table 2.** Habitual dietary intake and intake during each nutritional intervention

Condition	Energy intake (kcal)	Carbohydrate intake (g·kg^−1^·day^−1^)	Protein intake (g·kg^−1^·day^−1^)	Fat intake (g·kg^−1^·day^−1^)
Habitual	1622.4 ± 139.2	3.3 ± 0.8	1.1 ± 0.1	1.1 ± 0.1
GY	1892.2 ± 79.7	4.0 ± 0.3	1.9 ± 0.1*	1.0 ± 0.1
CHO	1959.4 ± 122.2	5.2 ± 0.3^†^	1.0 ± 0.1	1.0 ± 0.1

Values are mean ± SE; habitual dietary intake assessed by food frequency questionnaire; Dietary intake during the Greek Yogurt (GY) and isocaloric carbohydrate (CHO) intervention conditions, including whole food supplements, assessed using diet record. * indicates significantly greater (*p* < 0.05) in GY than CHO and habitual. † indicates significantly greater (*p* < 0.05) in CHO is than GY and habitual.

In order to determine differences in performance variables at the start of the training weeks due to a learning effect of multiple exposures to the performance battery, pre-training values were analyzed irrespective of supplement using a paired-sampled *t*-test. No differences at pre-training were noted (irrespective of supplement) for all performance variables, indicating no learning or order effect. The 5-day simulated soccer training camp was successful at inducing cumulative fatigue, leading to decrements in performance (Table 3). There were main effects of time observed for beep-test distance (*F* = 9.11, *p = *0.006, _p_*η^2^* = 0.27), CMJ height (*F* = 14.8 *p* = 0.01, _p_*η^2^* = 0.57), and BJ distance (*F* = 5.10, *p* = 0.04, _p_*η^2^* = 0.31), in that the values from pre-to-post intervention decreased, with no effect of intervention or intervention-by-time interactions (Table 3). All other performance variables, including 10 m and 20 m sprint, modified 5-0-5 Right, and Left did not change over time, nor was there an effect of intervention or intervention-by-time interaction.

**Table 3.** Participant performance data from pre- to post-training during each treatment condition

Variable	Pre	Post	∆ Change	Cohen’s *d*
Broad Jump Distance (cm) *				
GY	175.6 ± 5.4	171.8 ± 5.3	− 3.8 ± 2.1	0.20
CHO	177.7 ± 4.5	174.1 ± 4.0	− 3.6 ± 2.0	0.23
Counter-Movement Jump (cm) *				
GY	22.6 ± 1.0	22.0 ± 1.0	− 0.6 ± 0.2	0.18
CHO	22.8 ± 1.0	21.9 ± 1.1	− 0.84 ± 0.3	0.24
10 m Sprint (s)				
GY	1.85 ± 0.03	1.89 ± 0.04	0.04 ± 0.04	0.32
CHO	1.88 ± 0.05	1.87 ± 0.03	− 0.01 ± 0.05	0.07
20 m Sprint (s)				
GY	3.36 ± 0.05	3.46 ± 0.05	0.10 ± 0.04	0.56
CHO	3.42 ± 0.06	3.44 ± 0.05	0.02 ± 0.05	0.10
505 Right Foot Split (s)				
GY	3.06 ± 0.06	3.09 ± 0.04	0.03 ± 0.04	0.17
CHO	3.04 ± 0.05	3.09 ± 0.04	0.05 ± 0.02	0.28
505 Left Foot Split (s)				
GY	3.02 ± 0.06	3.07 ± 0.04	0.05 ± 0.04	0.28
CHO	3.03 ± 0.04	3.07 ± 0.04	0.04 ± 0.02	0.29
Beep-Test Distance (m)*				
GY	1270.7 ± 70.5	1136.9 ± 50.1	− 133.8 ± 34.4	0.61
CHO	1303.1 ± 72.0	1170.7 ± 47.8	− 132.3 ± 36.6	0.60

Performance data are presented as mean ± SE for pre- and post-training with each nutritional intervention Greek yogurt (GY) and isocaloric carbohydrate (CHO). Δ changes from pre- and post-training are presented as mean ± SE (95% CI). * indicates a significant time effect from pre- to post-training (*p* < 0.05).

**Figure 2.**
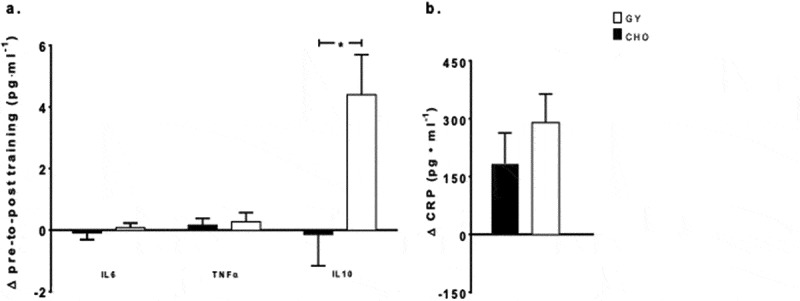
Changes in plasma concentrations from baseline (mean ± SE) of (a) interleukin 6 (IL6), tumor necrosis factor alpha (TNFα), and interleukin 10 (IL10) and (b) C-reactive protein (CRP), pre- to post-simulated soccer training camp with consumption of Greek Yogurt (GY) or isoenergetic carbohydrates (CHO), in adolescent female soccer players (cytokines, n = 13, CRP n = 11). * indicates a significant time-by-intervention interaction (*p* = 0.04) where GY showed a greater increase from pre to post than CHO.

Biochemical blood markers at pre-training were analyzed irrespective of nutritional intervention, using a paired-sampled *t*-test to determine if there were differences in physiological state at rest. No differences were detected at pre-training across all analytes (Table 4). There were no main effects for time or intervention, and no interactions observed for IL6, and TNFα (Figure 2) between the 5-day simulated training camps. IL10 showed a main effect for time (F = 8.32, *p = *0.014, _p_*η^2^* = 0.41) and a time-by-intervention interaction (*F* = 5.28 *p = *0.04, _p_*η^2^* = 0.31) (Figure 2a), reflecting an increase in IL10 from pre- to post-training with the consumption of GY (+26%, *p* = 0.008), but not with CHO. A main effect for time was observed for CRP (*F* = 16.6, *p = *0.005, _p_*η^2^* = 0.70), reflecting an overall increase from pre- to post-training (+65%, *p* = 0.005), with no intervention effect or interaction (Figure 2b).

**Table 4.** Resting levels of biomarkers pre-training

Biomarker	Condition	Mean ± SE	Significance
IL6 (pg·ml^−1^)	GY	2.6 ± 0.25	*p* = 0.86
	CHO	2.6 ± 0.34	
TNFα (pg·ml^−1^)	GY	4.6 ± 0.47	*p* = 0.92
	CHO	4.6 ± 0.45	
IL10 (pg·ml^−1^)	GY	16.7 ± 2.0	*p* = 0.58
	CHO	17.7 ± 2.3	
CRP (pg·ml^−1^)	GY	367.3 ± 116.9	*p* = 0.91
	CHO	355.3 ± 73.5	
IGF1 (ng·ml^−1^)	GY	346.6 ± 29.4	*p* = 0.29
	CHO	310.1 ± 35.7	
CK (u·L^−1^)	GY	66.9 ± 8.46	*p* = 0.58
	CHO	71.9 ± 7.96	

Values are mean ± SE; IL6, interleukin 6; IL10, interleukin 10; TNFα, tumor necrosis factor alpha; CRP, C-reactive protein; IGF-1, insulin-like growth factor-1; CK, creatine kinase.

**Figure 3.**
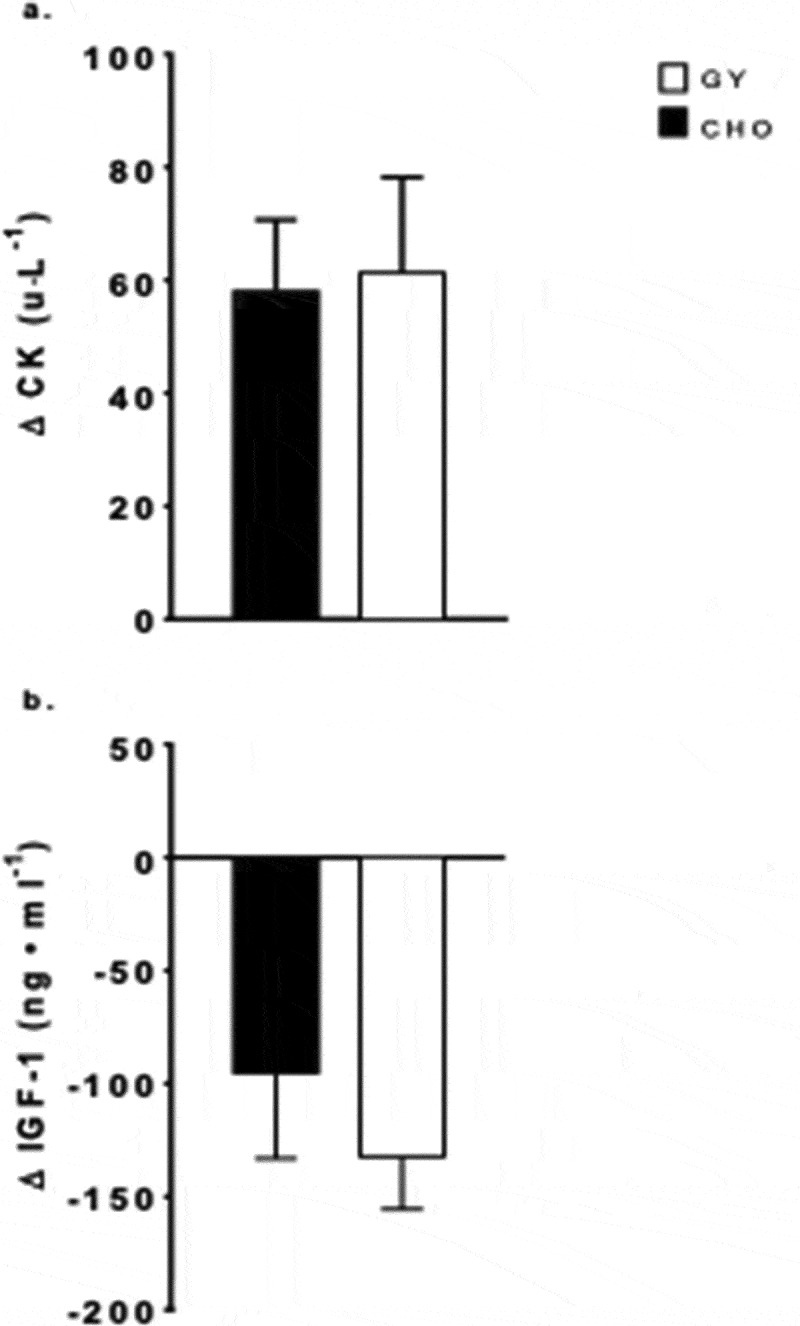
Changes in plasma concentrations from baseline (mean ± SE) of (a) creatine kinase (CK) and (b) insulin-like growth factor 1 (IGF-1), pre- to post-simulated soccer training camp with consumption of Greek Yogurt (GY) or isoenergetic carbohydrates (CHO), in adolescent female soccer players (CK *n* = 13, IGF-1 *n* = 13).

A main effect for time (*F* = 25.3, *p = *0.001, _p_*η^2^* = 0.68) was found for CK, which increased over time (+119%, *p ≤ *0.001), with no intervention effect or interaction (Figure 3a). Lastly, there was a main effect of time for IGF-1 (*F* = 20.1, *p = *001, _p_*η^2^* = 0.65), which was lower post-training compared to pre-training (−34%, *p ≤ *0.001), with no significant intervention effect or interaction (Figure 3b).

## 4. Discussion

Under certain circumstances, when the duration of recovery is inadequate due to a period of intensified exercise (e.g. training/identification camps), maintaining peak performance can become challenging due to an accumulation of fatigue. In the present study, we tested whether the regular provision of protein via GY, compared with an isoenergetic CHO control, would counter the declines in performance and enhance physiological recovery following a 5-day simulated intensified training camp in female adolescent soccer players. Despite the increased protein intake with GY, intense training resulted in similar decrements in performance, irrespective of nutritional intervention (i.e. GY and CHO). Similarly, we observed no differences in the pro-inflammatory response (IL6, TNFα and CRP) to training between nutritional interventions. However, GY led to significantly higher post-training concentrations of the anti-inflammatory cytokine IL10, compared to isoenergetic CHO. Despite an augmented anti-inflammatory response, no differences between groups were observed in CRP nor CK concentrations in response to training. Together, these results suggest that GY offered no added benefit above that observed with isoenergetic CHO for recovery following the 5-day intensified training in female adolescent soccer players but may assist with the acute anti-inflammatory response.

The 5-day intensified soccer-specific training camp led to impaired performance with reductions in jump performance (i.e. CMJ, BJ) and intermittent aerobic endurance (i.e. beep-test), but not in sprinting or agility measures (i.e. 10 m sprint, modified 5-0-5) (Table 3). Despite employing a pattern of ingestion thought to maximize muscle protein synthesis (i.e. ingestion immediately post-exercise), while also limiting muscle protein breakdown (i.e. consumption prior to bed and between breakfast and lunch the following day), the regular provision of GY did not provide any ergogenic benefit beyond that observed with the consumption of isoenergetic CHO. This occurred despite receiving a significant increase to their relative daily protein intake (from 1.1 to 1.9 g·kg^−1^·day^−1^), which is greater than that recommended for youth athletes (e.g. 1.35–1.6 g·kg^−1^·day^−1^) [27]. Although there is a paucity of research in female adolescent athletes with respect to exercise and nutrition, the present results are consistent with previous studies in both adult male [8,9] and female athletes [28], where the consumption of dairy-based protein resulted in trivial to no ergogenic benefit in performance recovery over 24–96 h following intensified training or competition, when compared to isoenergetic CHO. Although consumption of dairy-based protein has previously been shown to facilitate protein synthesis and reduce protein breakdown, specifically in adults [16], these mechanisms did not counter acute performance decrements any better than isoenergetic CHO following a period of intensified exercise in our youth athletes.

Similar to our previous work in adolescent swimmers [18], we demonstrated a significant increase in the anti-inflammatory cytokine IL10 with the consumption of dairy-based protein. While no significant changes were observed in pro-inflammatory cytokines IL6 and TNFα, CRP increased significantly with no difference between nutritional interventions following the soccer-specific intensified training camp. Although limited, a previous study in adults also reported increases in IL10 following acute intense exercise with the consumption of whey protein [19]. Together, these results suggest that post-exercise dairy-based protein consumption as a whole food or from isolated sources may elicit a transient anti-inflammatory response following acute and chronic exercise, which could be indicative of an anti-inflammatory function exerted by bioactive components of dairy-based proteins [29,30]. For example, dairy products contain essential nutrients, amino acids and other substances that can offer added antioxidant protection in response to increased oxidative stress (e.g. intense exercise) [30]. These effects can reduce activation of pro-inflammatory signaling pathways (e.g. nuclear factor kappa-B-signaling), reducing secondary injury from the subsequent immune response, expediting recovery [31]. Alternatively, it has been suggested that some amino-acids may exert direct effects on the immune cells altering cytokine expression [29]. Furthermore, while further research is required to tease out the exact mechanisms responsible for the augmented anti-inflammatory response, the observed increases in IL10 suggest an earlier polarization of immune cells to an anti-inflammatory phenotype (i.e. M1 to M2 macrophage), potentially reflecting earlier repair and regeneration processes. However, the augmented anti-inflammatory response observed in the present study did not translate into significant performance recovery. Future research should examine the effects of dairy-based protein consumption during a longer-duration training program.

High-intensity efforts combined with activities that incorporate eccentric movements (i.e. acceleration/deceleration and jumping) during competition and training elicit some degree of muscle damage [7]. This damage is characterized by the release of intramuscular proteins such as CK, which appears to be directly related to the extent of sarcomere disruption or skeletal muscle injury, and generally peaks ~24–48 h following exercise [7,32]. It has been previously suggested that estrogen may provide a protective effect against exercise-induced muscle damage [33] and as a result, females may have an attenuated CK response. In the present study, we controlled for circulating levels of estrogen by separating testing and training periods by 28 days to match the phase of the menstrual cycle. Indeed, there were no differences in pre-training estradiol concentrations between the two training weeks. Our simulated soccer-specific intensified training camp led to a significant increase in CK concentrations ~12 h following the final training session that was similar in magnitude to previously observed increases 48 h following an eccentric muscle damaging protocol in adolescent boys [34]. However, no difference in responses was detected between nutritional interventions. This CK response, along with the response to other dairy-based protein supplementations, is similar to that seen in both adult male and female athletes 24–48 h following chronic team-sport exercise and competition [9,28]. However, following acute eccentric exercise, Cockburn et al. [35] did observe an attenuation of CK 48 h post exercise with the consumption of milk-based protein. It is possible that dairy-based protein consumption reduces muscle damage via reduction of secondary injury, mediated by immune cells that may require a period of long passive recovery. However, complete rest or exercise cessation during a period of intensified training or competition may not be feasible due to the presence of performance assessments or scheduled match fixtures.

One limitation to our study is the lack of a direct measure of energy balance. The dietary information from the self-reported food records on training days, indicated that our participants’ energy intake (Table 2) was lower than dietary recommendations for youth athletes (e.g. ~2200 kcal·day^−1^), suggesting participants may have been in an energy deficit during both training weeks. However, these tools have previously been shown to result in an underestimation of energy intake in athletes [36,37]. Therefore, an energy deficiency cannot be determined by self-reported scales alone. Other indicators of an energy deficient state include weight loss, impairment of anabolic hormones (e.g. insulin, growth hormone, and IGF-1) and underperformance [38,39]. There was no change in body mass pre-to-post training, irrespective of nutritional intervention, which suggests that our participants were in energy balance. In fact, our participants’ body mass increased (non-significantly) during treatment with both interventions. However, IGF-1 concentrations declined pre-to-post training in our participants during both interventions, which could indicate an energy deficiency [38]. A transient decrease in IGF-1 in an energy balanced state has been associated with a transient increase in energy expenditure and an increase in pro-inflammatory markers, as observed in our athletes with the CRP post-training [40]. Future studies are needed with direct measures of energy expenditure to confirm the energy status of participants and how this might impact the effectiveness of the provision of dairy protein post exercise.

## 5. Conclusion

In summary, these results demonstrate that the provision of GY did not offer any added recovery benefit with respect to performance or in the attenuation of exercise-induced muscle damage above that achieved with an energy-matched carbohydrate supplement following 5-days of intensified soccer-specific training in female adolescent soccer players. However, regular consumption of GY may benefit the acute inflammatory response through an augmented anti-inflammatory response. Future research is required to further elucidate the role of GY as a potential training aid during a longer period of training, in mediating inflammatory processes and potential performance adaptations in youth athletes.

## Data Availability

The datasets used and/or analyzed during the current study are available from the corresponding author on reasonable request. The data are not publicly available due to REB restrictions.
